# Supplementary material for: Inflammatory Agonists Modulate the Host Response to Type 2 ECM scaffold Immune Environment and Long-Term Remodeling After Severe Traumatic Injury
Source: bioRxiv. 2025 Nov 11:2025.11.09.687444. Preprint. [Version 1] doi: 10.1101/2025.11.09.687444 (PMC12642640; doi:10.1101/2025.11.09.687444)
Supplement: Supplement 1 [file media-1.pdf]

Supplementary Information

Inflammatory Agonists Modulate the Host Response to Type 2 ECM scaffold Immune Environment and Long-Term Remodeling After Severe Traumatic Injury

Weizhen Li[1,a], Iris Baurceanu[1,a], Sanjay Pal[1,a], Rohan Chaudhari[3], Adrienne E. Kimmel[2], Matthew T. Wolf[1]*

[1]Cancer Biomaterials Engineering Section, Cancer Innovation Laboratory, Center for Cancer Research, National Cancer Institute, Frederick, MD 21702, USA

[2]Cancer Innovation Laboratory, Center for Cancer Research, National Cancer Institute, Frederick, MD 21702, USA

[3]Department of Cell, Developmental and Cancer Biology & Knight Cancer Institute, Oregon Health and Science University, Portland, OR 97201, USA

[a]Denotes equal contribution to this work.

*Correspondence to: matthew.wolf@nih.gov

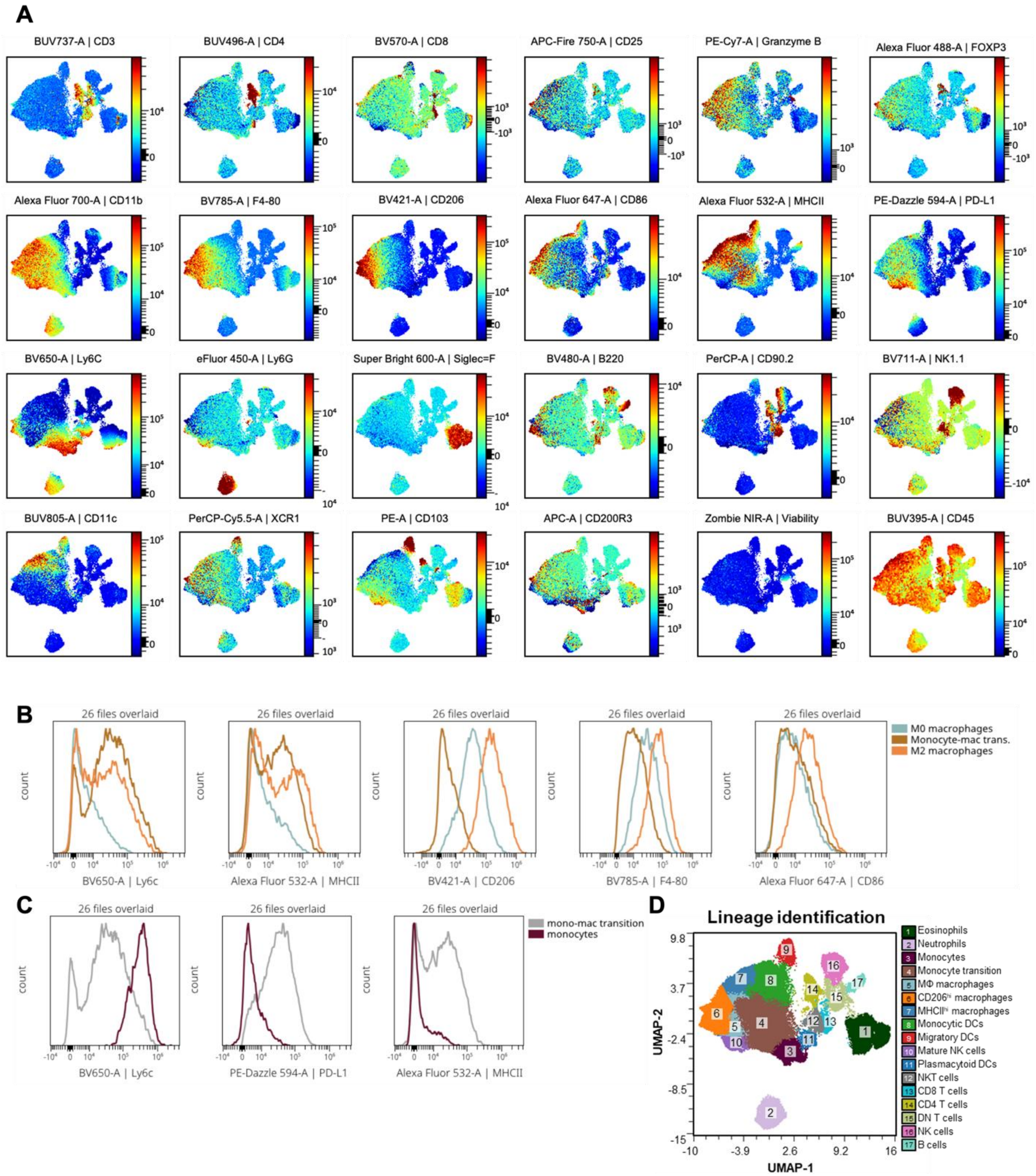

SFigure 1: UMAP analyses. (A) Week-1 UMAP with color coding the markers' expression level among all cells. Samples from all treatment groups were overlayed. **(B)** Histograms indicating the

expression profile differences among three macrophage subpopulations, M0, M2 and transitional macrophages. **(C)** Differentially expressed markers between monocytes and transitional macrophages. **(D)** Meta clustered UMAP.

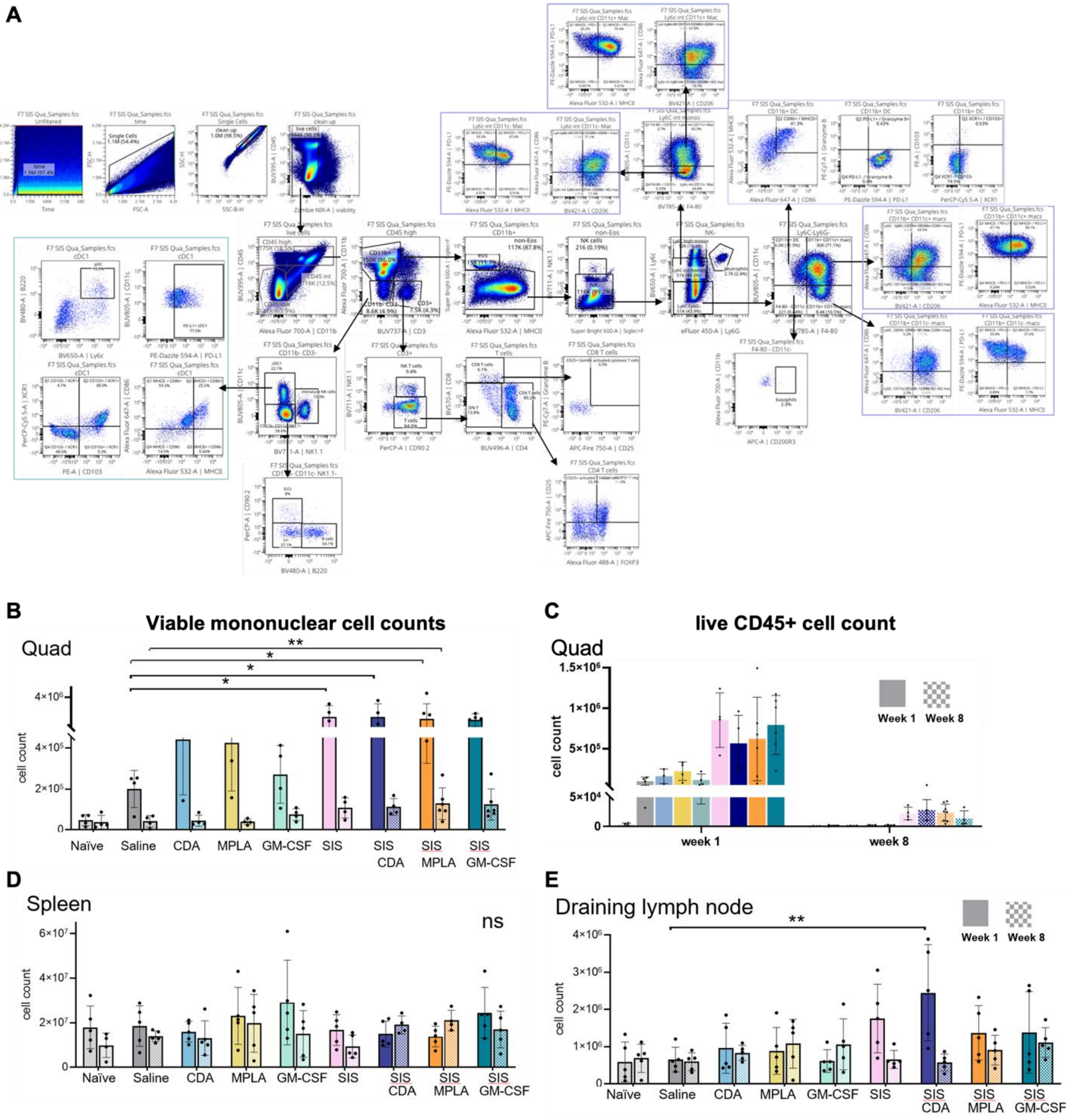

SFigure 2: (A) Flow cytometry gating strategy. Viable mononuclear cell counts from AOPI staining after tissue harvesting from **(B)** quadricep, **(D)** spleen and **(E)** draining lymph node at week 1 and 8. **(C)** Live CD45+ cell count from flow cytometer at week 1 and 8.

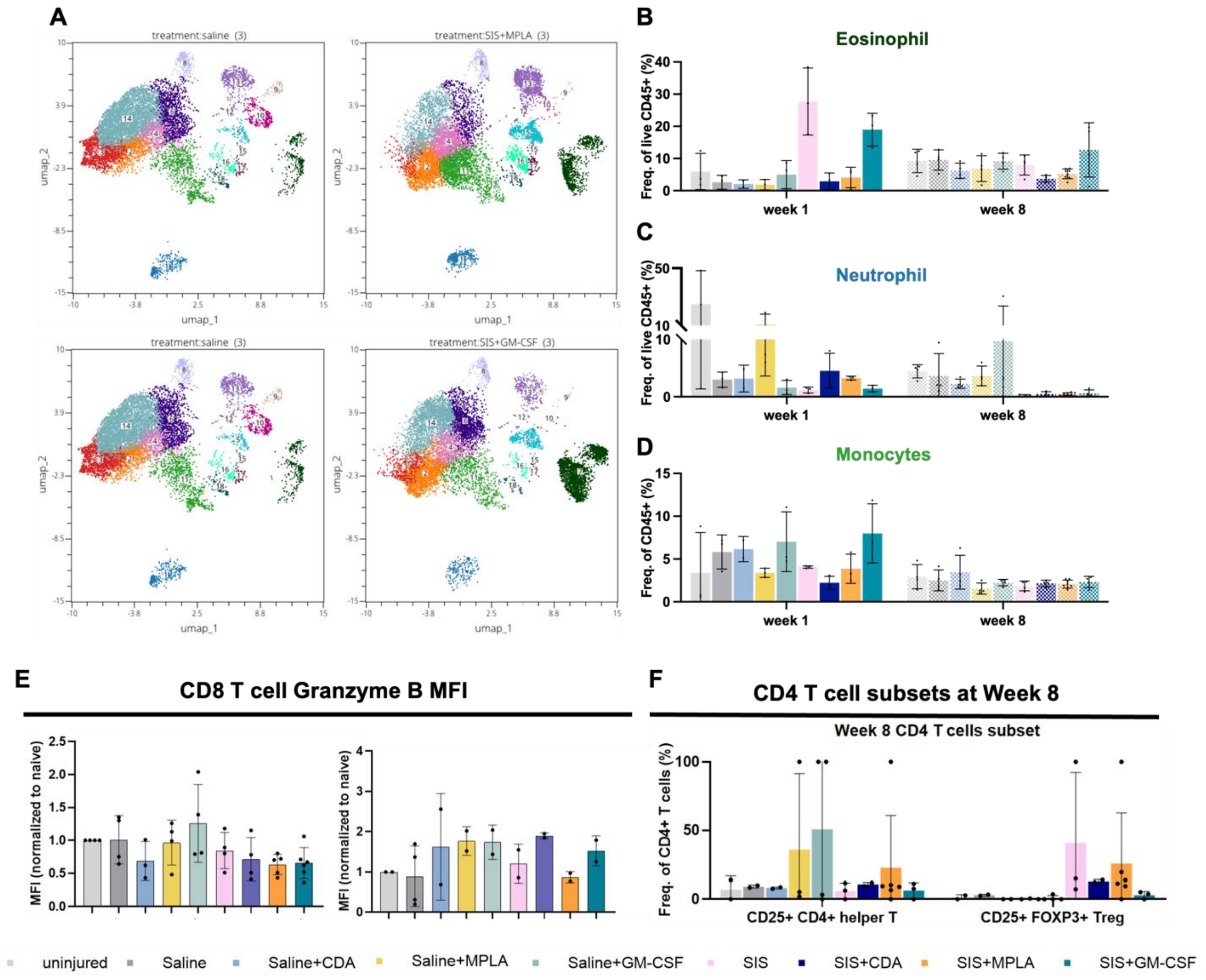

SFigure 3: (A) Clustered UMAP comparison among treatment groups. Percentage comparison of **(B)** eosinophils, **(C)** neutrophils and **(D)** monocytes among tr treatment groups at week 1 and 8. **(E)** Granzyme B mean fluorescent intensity comparison of CD8 T cells among treatment groups at week 1 and 8. **(F)** CD4 T cell activation state comparison at week 8.

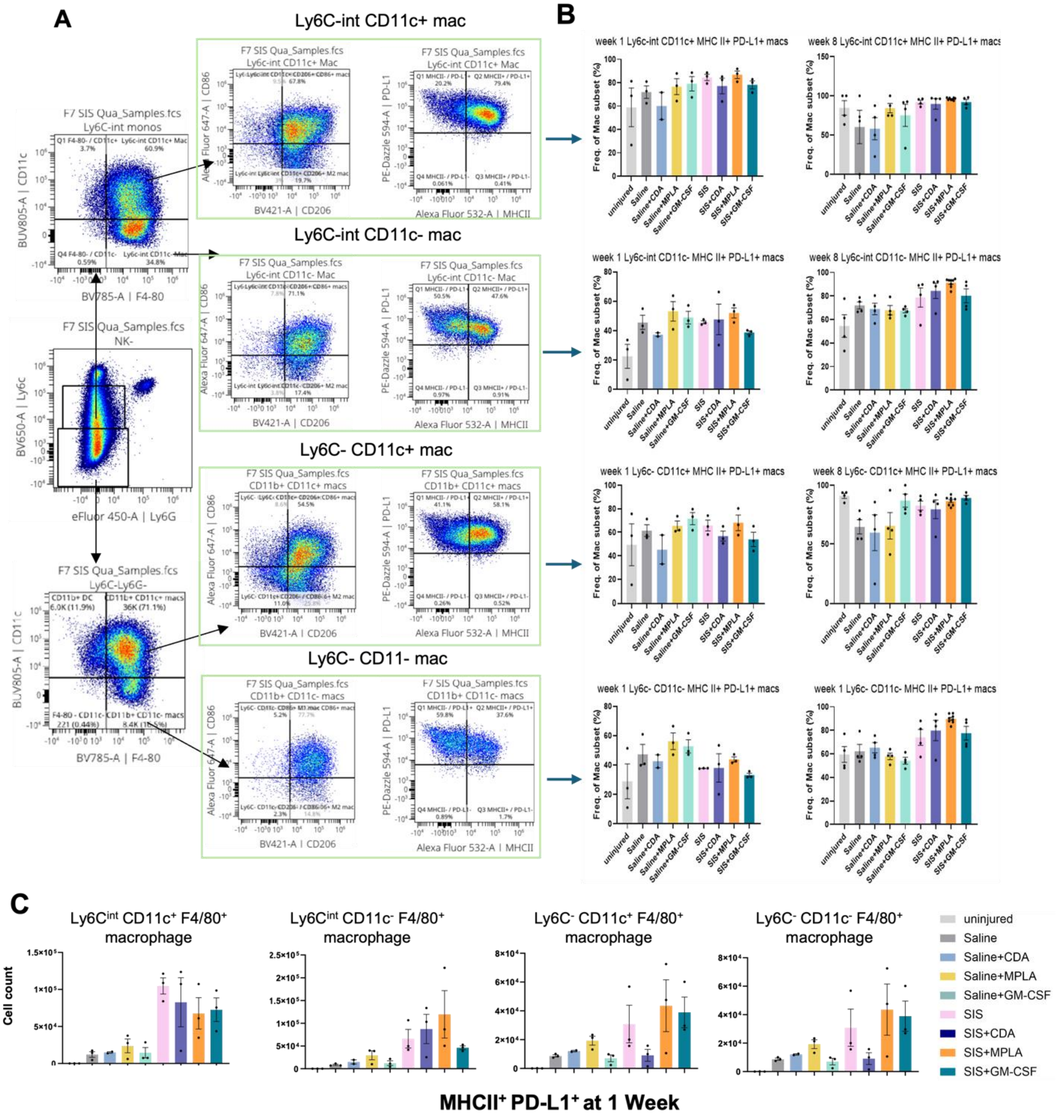

SFigure 4: (A) Macrophage subsets gating. **(B)** Macrophage subsets comparison in MHCII and PD-L1 expression levels at week 1 and 8. Comparison was made as percentage of macrophage subset. **(C)** Macrophage subsets cell count comparison at week 1.

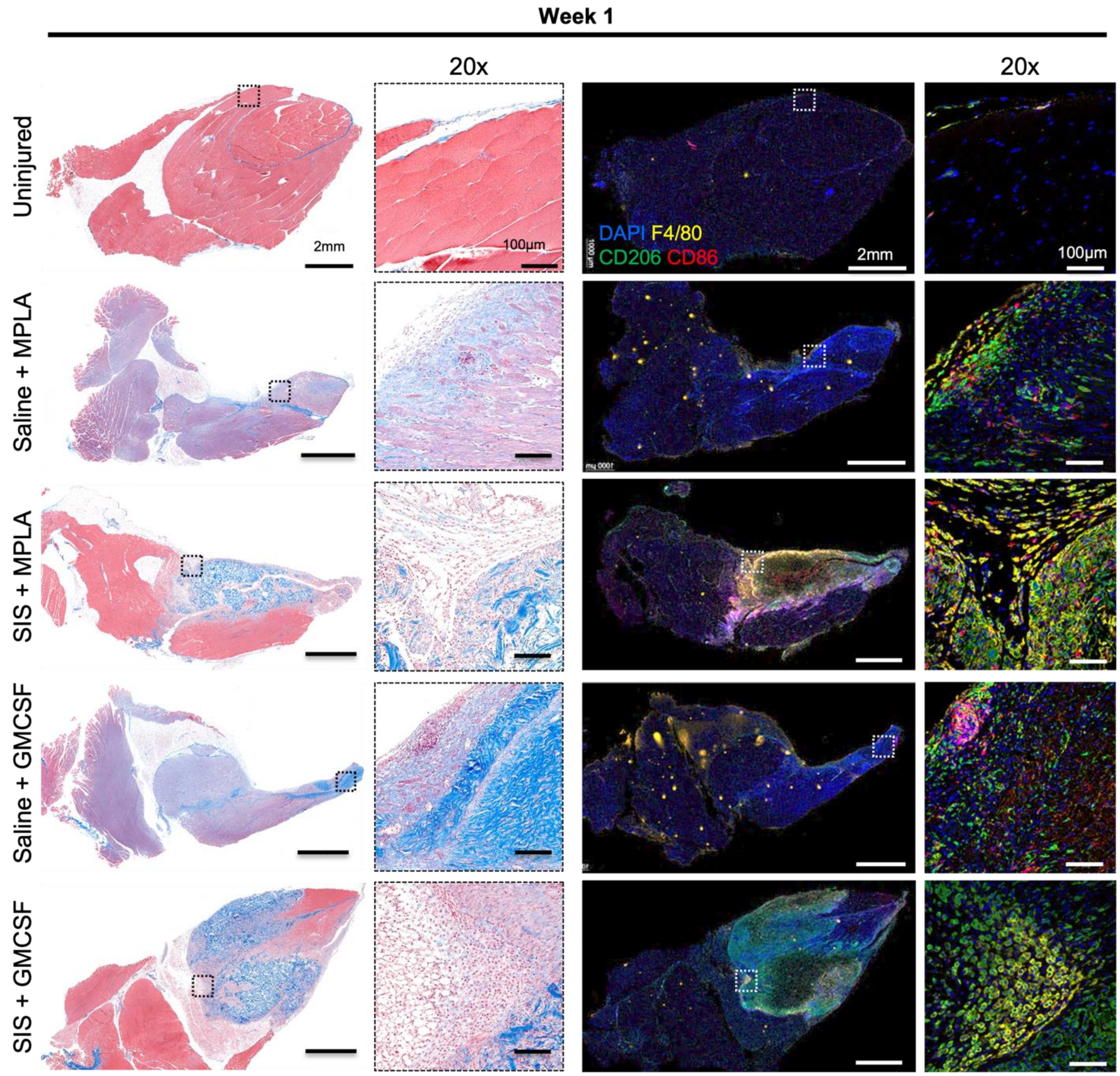

SFigure 5: Histologic and spatiotemporal immune profiling of VML injury post week 1 of ECM scaffold implantation. Masson's Trichrome-stained images (Left panel) and multiplex immunofluorescent images (Right panel) showing the morphology of VML injury microenvironment with immune agonist co-deliver (MPLA, and GM-CSF) with SIS-ECM scaffold or saline post 1 week of injury and implantation in C57Bl/6 mice quadriceps. Boxes highlight immune infiltrates at the at 20X objective.

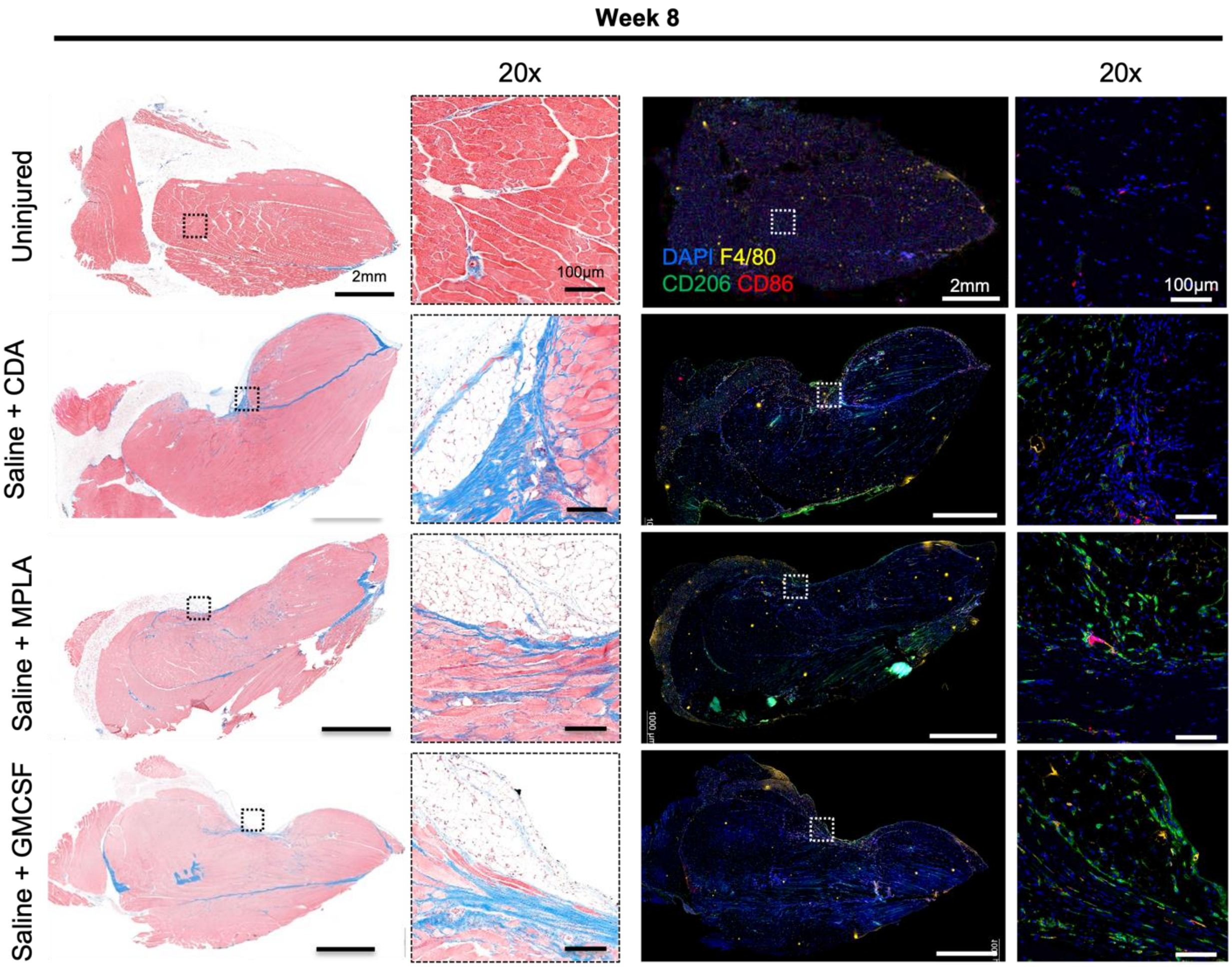

SFigure 6: Histologic and spatiotemporal immune profiling of VML injury post week 8 of ECM scaffold implantation. Masson's Trichrome-stained images (Left panel) and multiplex immunofluorescent images (Right panel) showing the morphology of VML injury microenvironment with immune agonist co-deliver (CDA, MPLA, or GM-CSF) with saline post 8 weeks of injury and implantation in C57Bl/6 mice quadriceps. Boxes highlight immune infiltrates at the at 20X objective.

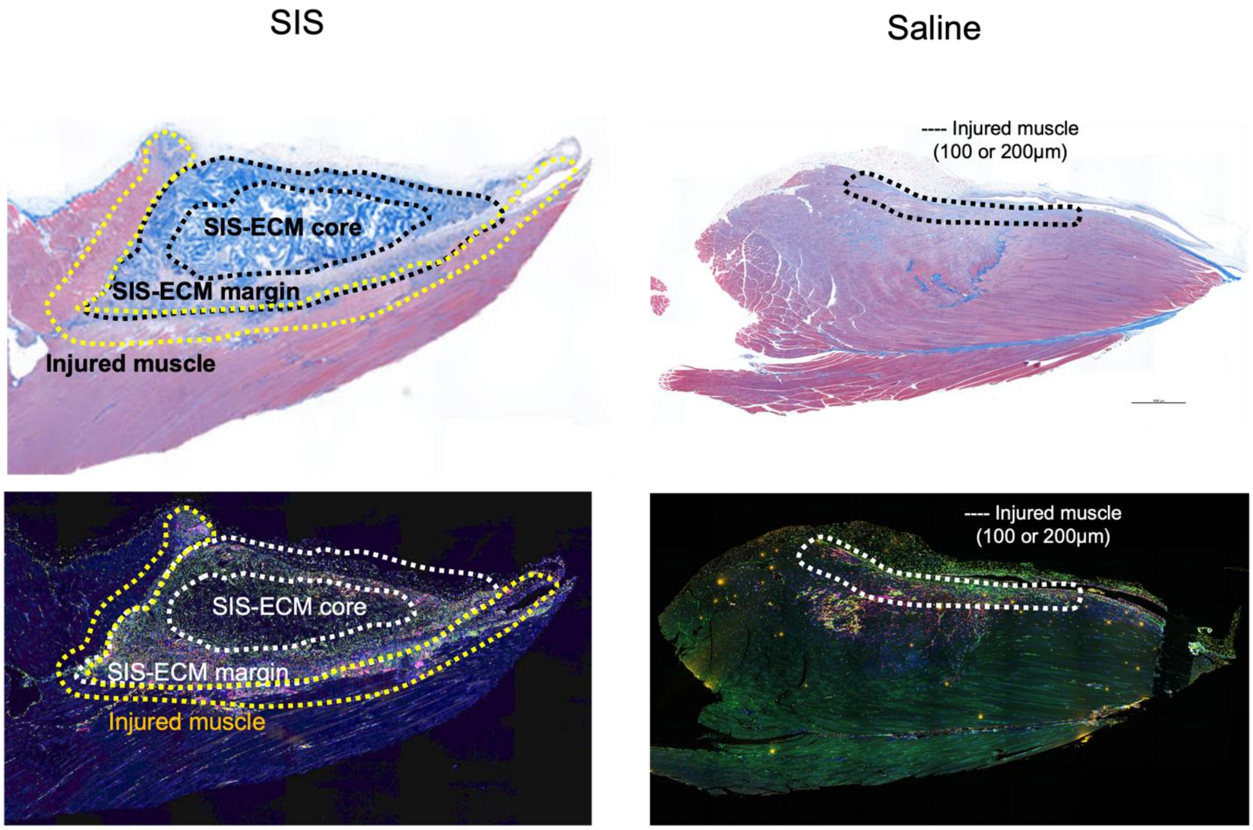

SFigure 7: Schematics showing the image annotation for cell quantification. Masson's Trichrome-stained images and multiplex immunofluorescent images showing the annotation used for quantification of immune cells in different region of the quadriceps muscle post injury and implantation with SIS-ECM scaffold or saline. The saline group has only injured muscle region (200µm from injury site towards the muscle) whereas the SIS-ECM scaffold group has three regions as marked in the image above showing the injured muscle region followed by SIS-ECM margin (200µm from injury site towards ECM) and there is a SIS-ECM core at the center of the scaffold.

Table 1: Antibodies used with dilution, clone no., Cat# and Company.

Target	Conjugate	Clone	species	Dilution	Company, Cat #
CD206/MRC1	-	E6T5J	rabbit	1:400	Cell Signaling Technology, Cat# 24595T
F4/80	-	BM8	rat	1:500	BioLegend, Cat# 123101
CD86	-	E5W6H	rabbit	1:500	Cell Signaling Technology, Cat# 20018
Anti-rabbit IgG	HRP polymer	-	-	Neat	Biocare Medical, REF# RMR622H
Anti-rat IgG	HRP polymer	-	-	Neat	Biocare Medical, REF# BRR4016H

Table 2: Antibodies used with dilution, clone no., Cat# and Company.

Fluorophore	Wavelength (nm)	Dilution	Manufacturer, Catalog #
Opal 570	550/570	1:150	Akoya Biosciences, #FP1488001KT
Opal 650	627/650	1:500	Akoya Biosciences, #FP1496001KT
Opal 520	494/525	1:150	Akoya Biosciences, #FP1487001KT
